# Betamethasone prevents human rhinovirus- and cigarette smoke- induced loss of respiratory epithelial barrier function

**DOI:** 10.1038/s41598-018-27022-y

**Published:** 2018-06-26

**Authors:** Eva E. Waltl, Regina Selb, Julia Eckl-Dorna, Christian A. Mueller, Clarissa R. Cabauatan, Thomas Eiwegger, Yvonne Resch-Marat, Katarzyna Niespodziana, Susanne Vrtala, Rudolf Valenta, Verena Niederberger

**Affiliations:** 1 Department of Otorhinolaryngology, Medical University of Vienna, Vienna, Austria; 2 Department of Pathophysiology and Allergy Research, Center for Pathophysiology, Infectiology and Immunology, Division of Immunpathology, Medical University of Vienna, Vienna, Austria; 3 Division of Immunology and Allergy, Food allergy and Anaphylaxis Program, The Department of Paediatrics, The Hospital for Sick Children, Toronto, Canada; 4 Research institute, The Hospital for Sick Children, Translational Medicine program, Toronto, Canada; 5 Department of Immunology, The University of Toronto, Toronto, Canada; 6 NRC Institute of Immunology FMBA of Russia, Moscow, Russia

## Abstract

The respiratory epithelium is a barrier against pathogens and allergens and a target for therapy in respiratory allergy, asthma and chronic obstructive pulmonary disease (COPD). We investigated barrier-damaging factors and protective factors by real-time measurement of respiratory cell barrier integrity. Barrier integrity to cigarette smoke extract (CSE), house dust mite (HDM) extract, interferon-γ (IFN-γ) or human rhinovirus (HRV) infection alone or in combination was assessed. Corticosteroids, lipopolysaccharide (LPS), and nasal mucus proteins were tested for their ability to prevent loss of barrier integrity. Real-time impedance-based measurement revealed different patterns of CSE-, HDM-, IFN-γ- and HRV-induced damage. When *per se* non-damaging concentrations of harmful factors were combined, a synergetic effect was observed only for CSE and HDM. Betamethasone prevented the damaging effect of HRV and CSE, but not damage caused by HDM or IFN-γ. Real-time impedance-based measurement of respiratory epithelial barrier function is useful to study factors, which are harmful or protective. The identification of a synergetic damaging effect of CSE and HDM as well as the finding that Betamethasone protects against HRV- and CSE-induced damage may be important for asthma and COPD.

## Introduction

The respiratory epithelium represents a first line of protection against airborne particles, including pathogens and allergens, and plays an important role in the regulation of airway inflammation and host defense^1^. The nasal and the bronchial epithelium are naturally exposed to a variety of different potentially damaging factors, which may injure the integrity of the mucosa by different mechanisms. Infections with respiratory viruses, exposure to cigarette smoke and/or exhaust fume as well as to protease-containing bioparticles and endogenous inflammatory factors are among the commonly encountered factors offending the respiratory tract^2,3^.

Cigarette smoke, virus infections, house dust mites (HDMs) and cytokines all cause impairment of epithelial integrity by different mechanisms. Cigarette smoke has been shown to affect tight junction (TJ) organisation of airway epithelial cells directly by cleaving occludin and claudin and indirectly through oxidative stress, which leads to the production of cytokines (interleukins IL-6 and IL-8) in epithelial cells, which in turn cause airway inflammation^4^. Human rhinoviruses (HRV) are the most commonly encountered respiratory viruses and are the most frequent cause of common cold infections and asthma exacerbations^5^. Allergen-containing extracts (e.g., HDMs, *Alternaria alternata*) have been shown to either cause damage directly or to act in a synergetic manner with other damaging factors (e.g., rhinovirus) on airway epithelium^6,7^. Pro-inflammatory cytokines, such as interferon-γ (IFN -γ) and IL-4 are endogenously secreted during chronic allergic inflammation and were reported to impair epithelial barrier function^8,9^.

Tissue culture models based on respiratory airway cell lines have been useful to investigate the influence of factors affecting epithelial barrier function under controlled experimental conditions^9–11^. In this study we established an *in vitro* model for the assessment of respiratory epithelial barrier function based on primary nasal epithelial cells and a real-time impedance-based platform for measurement of barrier function to assess some of the most important damaging factors for respiratory epithelial barrier function. In particular we compared the isolated and synergetic effects of cigarette smoke extract (CSE), HRV infection, HDM and IFN-γ on primary nasal epithelial cells in comparison with the human bronchial epithelial cell line 16HBE14o-. Furthermore, we tested the effects in the traditional transwell system as well as in a real-time impedance-based system. Our study demonstrates a strong synergistic effect of CSE and HDM-extract regarding epithelial barrier damage and we observed that the steroid Betamethasone protected against HRV and CSE-induced damage. These findings may have important implications for the treatment of HRV-induced asthma and chronic obstructive pulmonary disease (COPD).

## Results

### Characterisation of primary human nasal epithelial cell cultures

Primary cells were obtained from adult subjects from the inferior nasal turbinate during routine surgery. On average 2.6 × 10^7^ (SD ± 2.2 × 10^7^) cells were isolated per subject. After 21 days of air-liquid interface (ALI) culture, histological assessment of cells prepared by cytospin showed normal morphology with apical cilia (Supplementary Fig. S1). Normal ciliary beat frequency (12–14 Hertz) was confirmed by microscopic video analysis (Supplementary video). The epithelial nature of cultured cells was additionally assessed by flow cytometry analysis (pan-cytokeratin positive and CD45 negative cells; data not shown). Primary nasal epithelial cells from allergic and non-allergic subjects showed no relevant differences regarding several parameters (data not shown). In detail, we checked the time until they formed confluent monolayers by microscopic observation, checked the tightness of the layer by measuring transepithelial resistance (TER)^12^ and their ability to regain confluency after physical damage^13^.

### Measurement of different types of damage of epithelial cell barrier function by the transwell and the impedance-based xCELLigence system

**Figure 1:**
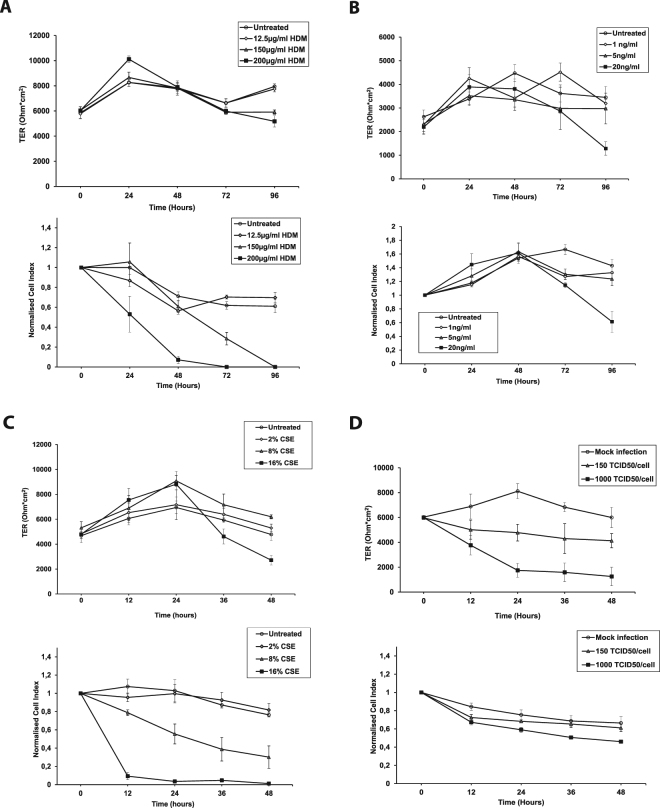
Comparison of two different methods to determine the influence of HDM extract, interferon-γ (IFN-γ), cigarette smoke extract (CSE) and human rhinovirus 14 (HRV 14) infection on barrier function in primary nasal epithelial cells. Primary human nasal epithelial cells were grown in a 2-chamber tissue culture model (A–D, top panels) or on E-plates in the xCELLigence system (A–D, bottom panels). Changes of trans-epithelial resistance (TER, y-axes, top panels) or of cell impedance (normalised Cell Index, y-axes, bottom panels) were determined at indicated time points (x-axis) after exposure of cells to different concentrations of HDM extract (**A**) IFN-γ (**B**) CSE (**C**) or HRV 14 (**D**). Results from three independent experiments performed in duplicates are shown. Statistical significance analyses were performed with the two-sided Welch-tests. Standard errors of the mean values are displayed as error bars for each measuring point.

Primary human nasal epithelial cells were cultured as confluent monolayers on a semipermeable membrane in the transwell system and on E-plates in submerged culture in the label-free impedance-based xCELLigence system (Fig. 1). After reaching TER values of at least 1000 Ohm * cm^2^ (transwell system) or a minimum absolute Cell Index of 12 (xCELLigence system), cell monolayers were exposed to different concentrations of HDM extract (Fig. 1A), IFN-γ (Fig. 1B), CSE (Fig. 1C) or HRV 14 (Fig. 1D). Upon exposure to damaging factors TER values and impedance values decreased quite comparably in a time- and dose-dependent manner.

However, a few interesting differences were observed in the two systems (Fig. 1A–D). Culture of primary cells in the impedance-based system, which integrates a measure of viability, morphology and thickness of the epithelium, was more sensitive to show the cell damaging effect after exposure of cells to HDM proteins (Fig. 1A lower panel). Here, a decrease of the Cell Index was already visible at 200 µg/mL of HDM extract within the first 24 hours, while an equal concentration led to a change of TER values only after 96 hours compared to the negative control (Fig. 1A upper panel). Exposure to 12.5 µg/mL of HDM extract, a concentration which is known to already induce positive reactions in nasal provocation tests in HDM allergic patients, did not influence the barrier function of the cells in either culturing system.

Cells were exposed to IFN-γ via the basolateral compartment in the transwell system and via the apical side in the impedance-based system. In both cases, an impairment of barrier function as compared to the negative control was seen only after several days of exposure (72 hours; Fig. 1B). The xCELLigence system is proved to be more sensitive in showing the effect of low concentrations of the cytokine (i.e., 1 ng/mL, 5 ng/mL), while exposure to 20 ng/mL was necessary to induce a drop of TER in the transwell system (Fig. 1B). Addition of standardised CSE to the cells led to the fastest change of Cell Index values in comparison to the other tested factors (Fig. 1C, lower panel). Already after 12 hours of incubation with 8% of CSE, a substantial decrease of Cell Index was observed. In contrast, resistance values in the transwell system decreased only at a concentration of 16% CSE and after 36 hours of exposure (Fig. 1C, upper panel). HRV 14 infection led to a decrease of barrier function at 150 TCID50/cell after 12 hours both in the transwell system and in the xCELLigence system, but the difference compared to non-infected cells was more pronounced in the transwell system.

For control purposes, we tested all concentrations of the four damaging factors used in this study for their cytotoxic effect using the crystal violet assay. No cytotoxic reaction of cells was observed when they were exposed to up to 200 µg/mL HDM extract, 20 ng/mL of IFN-γ or up to 6 µg/mL Betamethasone for 96 hours, or with up to 16% CSE for 48 hours, or 1000 TCID50/cell RV14 (Supplementary Fig. S3A–E).

Additionally a quantitative cell viability assay was performed (Supplementary Fig. S5). These results show that none of the substances have a relevant cytotoxic effect on the cells as shown with the crystal violet assay. For comparison (i.e., positive control), cell death was induced with ethanol.

### Primary nasal epithelial cells for evaluation of factors affecting epithelial barrier function in comparison to 16HBE14o- cells

**Figure 2:**
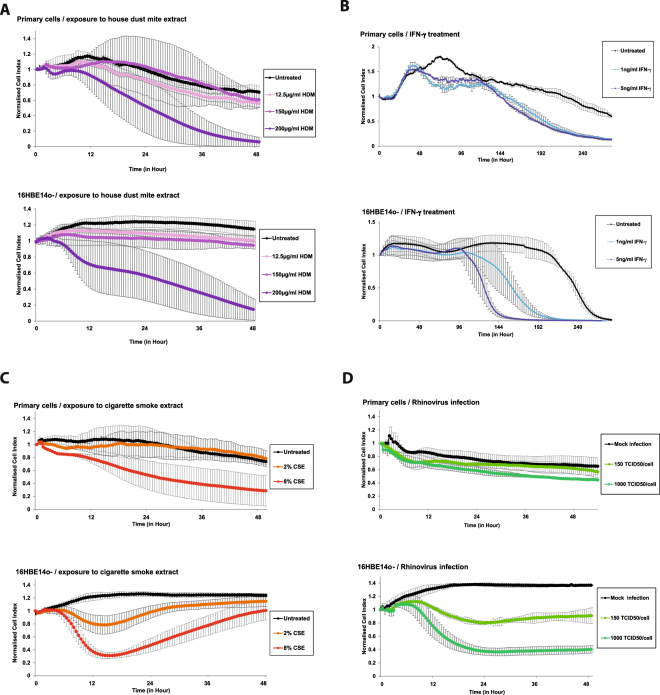
Effect of four different damaging factors on primary human nasal epithelial cells and on the respiratory epithelial cell line 16HBE14o-. Primary cells (top panels) and the respiratory cell line (bottom panels) were exposed to different concentrations of HDM (**A**) interferon-γ (**B**) standardised cigarette smoke extract (**C**) or human rhinovirus 14 (**D**). Data were normalised at the time of addition of factors (time point 0). Impedance values were measured every 30 minutes by the xCELLigence system for 48 hours (**A**,**C**,**D**) or 240 hours (**B**) (x-axes) and are expressed as a normalised Cell Index (y-axes). Results are derived from three independent experiments which were done in duplicates. Statistical significance analyses were performed with the two-sided Welch-tests. Standard errors of the mean values are displayed as error bars for each time point in (**A**,**C**,**D**) while in (**B**) the standard error of the mean is shown only for every 5^th^ measuring point for better visualisation.

As a next step, we evaluated whether the effects seen after exposure of primary cells to the four different factors were similar in 16HBE14o- cells. 16HBE14o- cells express tight junctions and functional cilia and are used as surrogates to test respiratory epithelial barrier function^14^. We observed that HDM (Fig. 2A), IFN-γ (Fig. 2B), CSE (Fig. 2C) and HRV 14 infection (Fig. 2D) all led to a dose dependent decrease of barrier function in primary nasal epithelial cells (Fig. 2, top panels). However, 16HBE14o- cells were more sensitive to damage because lower concentrations of HDM extract (primary cells: 200 µg/mL; cell line: 12.5 µg/mL), CSE (primary cells: 8%, cell line: 2%) and HRV 14 (primary cells: 1000 TCID_50_/cell, cell line: 150 TCID_50_/cell) already induced decreases of the Cell Index (Fig. 2, bottom panels). Furthermore, the damaging effects of CSE for cell integrity were observable earlier in 16HBE14o- cells (Fig. 2C, bottom panel).

### Synergetic damaging effect of HDM extract and CSE on respiratory epithelial cells

**Figure 3:**
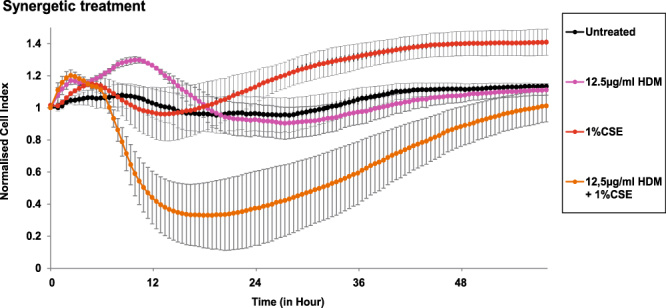
Synergetic effect of non-damaging concentrations of cigarette smoke and HDM extract on barrier function. 16HBE14o- cells grown in the xCELLigence system were exposed to either 12.5 µg/mL of HDM extract, or 1% of cigarette smoke extract, or a combination, or a medium control (untreated). Data were normalised at time point 0 and impedance values (y-axis: normalised Cell Index) were then measured every 30 minutes by the xCELLigence system for 48 hours (x-axis). Results are derived from three independent experiments performed in duplicates. Statistical significance analyses were performed with the two-sided Welch-tests. Standard errors of the mean values are displayed as error bars for each measuring point.

Next, we performed a detailed investigation regarding synergetic damaging effects of each of the damaging factors by combining HRV14 infection and HDM exposure, exposure to HDM and IFN-γ, CSE and HRV14, as well as HDM and CSE at concentrations for each factor, which *per se* did not reduce barrier function. Under these conditions a synergetic effect of damaging factors was only found for the combination of HDM and CSE exposure (Fig. 3). The combination of a low concentration of HDM extract (12.5 µg/mL) and standardised cigarette smoke extract (1%), which both alone did not lead to a decrease of the Cell Index, led to a substantial impairment of the cell monolayers. This effect was already visible 6 hours after addition of both factors (Fig. 3).

### Protective effect of the nasal steroid Betamethasone on epithelial barrier

**Figure 4:**
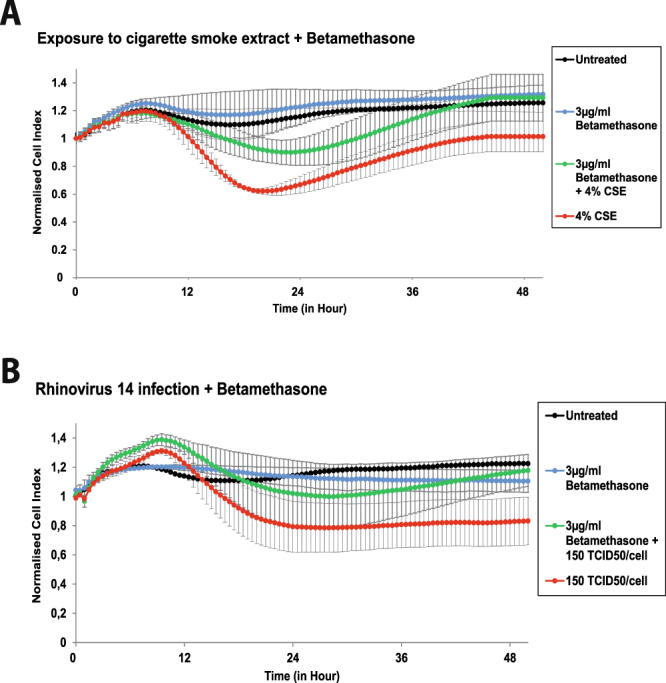
Betamethasone prevents cigarette smoke- and rhinovirus-induced damage of respiratory epithelial cells. 16HBE14o- cells were incubated at time point 0 with Betamethasone (3 µg/mL) or medium. Three hours thereafter, either 4% cigarette smoke extract (**A**) was added to the cells or cells were infected with 150 TCID_50_/cell of rhinovirus 14 (**B**). Data were normalised at time point 0 and impedance values (y-axis: normalised Cell Index) were measured every 30 minutes for 48 hours. Results from three independent experiments performed in duplicates are shown. Statistical significance analyses were performed with the two-sided Welch-tests. Standard errors of the mean values are visualised as error bars.

Next, we tested several substances regarding their protective effects on barrier function in respiratory epithelial cells, among them LPS^15^, abundantly expressed proteins in the nasal mucus of non-allergic subjects (e.g., haptoglobin)^16^ and nasal steroids^17^. We found modest effects of LPS on HDM-induced epithelial barrier damage and haptoglobin seemed to protect against CSE-induced damage (data not shown). However, the most consistent protective effect was found when cells were pre-treated with a single dose of Betamethasone (3 µg/mL) which protected against barrier damage induced by CSE (4%) exposure or against infection with HRV 14 (150 TCID_50_/cell) (Fig. 4A,B). The concentration of Betamethasone applied in these experiments corresponded to the daily recommended amount of commercially available nose drops (Betnesol®), containing Betamethasone as the active agent. When epithelial cells were incubated with 2.5 µg/ml Fluticasone propionate (daily recommended amount of commercially available nasal spray Flixonase®, containing Fluticasone propionate as the active agent) no protective effect was observed (Fig. S4A and B). Also when cells were pre-treated with Betamethasone and were subsequently exposed to IFN-γ or HDM, no inhibition of damage was observed (data not shown).

## Discussion

The respiratory epithelial barrier is important to protect the respiratory tract against the intrusion of noxious factors and allergens and is thus a prime target for therapy in respiratory allergy, asthma and COPD.

In order to investigate the effects of three major factors (i.e., HDM extract as an example for a protease-containing allergen extract, HRV and CSE) involved in respiratory allergy, asthma and COPD, we established a surrogate system for testing respiratory epithelial barrier function based on respiratory epithelial cells and real-time impedance measurement. We found that the impedance-based system is advantageous in enabling continuous measurement of cell responses over several days, thus allowing finer discrimination of small damaging effects. Substantially lower concentrations of damaging factors, except for infection with HRV, were necessary in the impedance-based platform to impair barrier function compared to the transwell system. The reason for this finding might be the continuous measurements and the high discrimination of small damaging effects in the xCELLigence system. Another advantage of the impedance-based platform is that measurements can be performed at defined temperature conditions whereas resistance values of the transwell system can only be measured at room temperature, so that the cells have to be cooled down at every measuring time point. When assessing respiratory epithelial barrier function with the real-time impedance measurement based on primary respiratory epithelial cells the results were comparable to the traditional transwell measurements, showing that the xCELLigence system reliably measures barrier function and its disturbance by the studied factors. Only for IFN-γ we noted that the transwell system seemed to be more sensitive than the xCELLigence system but this may be due to the fact that IFN-γ was added to the basolateral well in the transwell system which was not possible for the xCELLigence system.

Interestingly, we found no relevant differences regarding the formation of the epithelial barrier neither in the transwell nor in the impedance-based system depending on the origin of the primary nasal respiratory cells (i.e., obtained from allergic and non-allergic subjects), as it has been suggested in another recent study^17^. Regarding the investigation of the three exogenous damaging factors (i.e., HDM allergen extract, CSE and HRV) several important findings were made. In fact there are conflicting data regarding the barrier-damaging effect of HRV. One recent study reported that HRV infection does not have any effect on epithelial barrier function^18^, whereas other studies^7,10,12^ have shown that HRV infection indeed reduces barrier function. Our study unambiguously demonstrates that HRV infection impairs respiratory barrier function in the impedance-based as well as in the transwell system, regardless whether primary epithelial cells or epithelial cell line are used.

A barrier-disrupting effect of HDM extract, which has been attributed to the effect of proteases (e.g., Der p 1) has been reported^19^. However, in this study as well as in other studies investigating HDM extracts at non-physiological high concentrations of HDM extracts have been used^20^. We were able to observe a barrier decreasing effect with HDM extract, which was produced freshly from *D*. *pteronyssinus* containing faeces, only when using non-physiologic concentrations. The presence of Der p 1, which reportedly has protease activity, was detected in the HDM extract that we used (Supplementary Fig. S2). A synergetic barrier-disrupting effect was found using a concentration of 12.5 µg/mL HDM, when cells were simultaneously exposed to cigarette smoke extract (i.e., 1% of CSE). This HDM extract concentration of 12.5 µg/mL is substantially higher than concentrations of HDM commonly observed in bedding^21^. Concentrations in this range may be reached under conditions of nasal provocation experiments, which cannot be regarded as a physiologic exposure^22^. However, co-exposure to HDM and smoke or exhaust fumes may be important for the process of allergic sensitisation, which is supported by epidemiological studies^23,24^. Thus our study identifies HRV and CSE as major exogenous factors for the damage of the respiratory epithelial barrier. We therefore think that our finding that Betamethasone had a protective effect on barrier damage caused by HRV infection and CSE exposure is of high clinical relevance. In fact, clinical studies have shown that the use of nasal steroids as a treatment for acute rhinosinusitis shortens disease duration and decreases symptom severity^25^.

However, in our study only Betamethasone but not Fluticasone propionate exerted a protective effect on epithelial cells. Betamethasone and Fluticasone are both topically active corticosteroids and are both frequently used for the treatment of inflammatory diseases of the nose^26,27^. The concentrations of Betamethasone (3 µg/ml) and Fluticasone propionate (2.5 µg/ml) used by us corresponded to the recommended daily dose of commercially available nasal drops (Betnesol®) and nasal spray (Flixonase®). The daily recommended maximum dose of Fluticasone propionate is 400 µg (4 spray actions of 50 µg per nostril daily), the maximum recommended daily dose of Betamethasone is 540 µg (9 drops of 30 µg per nostril daily). To exclude potential differences in our model we used Fluticasone propionate and Betamethasone both as a pure substance. Suprisingly we found that only Betamethasone but not Fluticasone was able to suppress the damaging effect of 4% cigarette smoke extract or 150 TCID_50_/cell of rhinovirus infection. This difference can not be explained by a difference in the potency of the two drugs, as both of them belong to the same potency topical steroid groups^28^. However, Betamethasone and Fluticasone show different hydrophilicity/hydrophobicity which may explain their different effects.

Thus, our results indicate that the steroid Betamethasone may exert its therapeutic effect not only through its anti-inflammatory properties on immune cells but also through a reduction of epithelial damage caused by rhinovirus infection or cigarette smoke exposure. The fact, that we obtained a protective effect on CSE-induced damage only with Betamethasone but not with Fluticasone, a steroid currently used for treatment of COPD^29^, may be also important because it may rekindle the interest in Betamethasone as a possible treatment in COPD.

In summary, our results demonstrate that Betamethasone has a protective effect on CSE and HRV-induced respiratory epithelial damage. These findings may have important clinical implications because they indicate that Betamethasone may be useful in the treatment of HRV-induced asthma and of COPD.

## Methods

### HDM extract preparation

Aliquots of 160 µg HDM extract containing faeces from the HDM *Dermatophagoides pteronyssinus* (#4960 Allergon; Ängelholm, Sweden) were vortexed in 0.5 mL PBS at 4 °C for 5 min. Insoluble material in the protein extract was removed by centrifugation for 5 min at 4 °C with 4000 rpm. Protein concentration of the extracts (supernatants) was analysed and measured by BCA protein assay (Bradford BioRad; Hercules, USA). The presence of HDM allergens in the extract was studied with rabbit antibodies specific for nDer p 1, rDer p 2, rDer p 5, rDer p 7, rDer p 21 and rDer p 23 by immunoblotting (Supplementary Fig. S2)^30–34^. Serum from a non-immunised rabbit was used for control purposes. For immunoblotting, 1500 µg/mL (30 µg/cm) of *D*. *pteronyssinus* extract were separated by 12.5% SDS-PAGE (protein ladder PageRuler^TM^ Plus, Thermo Fisher Scientific; Waltham, USA; used as a molecular weight marker) and blotted onto a nitrocellulose membrane (Schleicher & Schuell, Dassel, Germany), which was afterwards cut into strips of 0.3 cm width. The strips were blocked in buffer A [50 mm sodium phosphate pH 7.5, 0.5% (w/v) BSA, 0.5% (v/v) Tween-20 and 0.05% (w/v) sodium azide] and incubated overnight at 4 °C with rabbit antisera at a dilution of 1:5000. After washing, the nitrocellulose membranes were incubated with ^125^I-labeled donkey anti-rabbit IgG antibodies diluted 1:10 000 (Perkin Elmer, Boston, MA, USA) and bound antibodies were detected by autoradiography (Kodak XOMAT film, Kodak, Heidelberg, Germany)^35^. The transfer membrane was scanned with the Photo Scanner Epson Perfection V370 Perfection (Seiko Epson Corporation, Japan) in 600 dpi.

### Preparation of standardised aqueous cigarette smoke extract

CSE standardised for its nicotine content (i.e., 44 ng nicotine/mL) was produced as previously described^36,37^. In short, two commercially available filter cigarettes (Marlboro, Philip Morris International Inc., New York, USA; nicotine: 0.8 mg; tar 10 mg) were consecutively bubbled through 8 mL of Minimum Essential Medium (MEM, Gibco; Thermo Fisher Scientific; Waltham, USA) to produce CSE^37,38^. To mimic the smoking habits of an average smoker the device smoked cigarettes at a rate of 15 mL/sec for 2 sec long followed by a 28 sec pause^38^. The resulting nicotine concentration is comparable with plasma nicotine concentrations of average smokers (43.7 ± 38 ng/mL)^39^. The dilutions used in this study (1–32% CSE, produced by the cigarette smoking machine) were in the range of 14.4 (32%) to 0.45 (1%) ng nicotine/mL.

### Rhinovirus preparation

Human rhinovirus 14 (HRV14; ATCC, The Global Bioresource Center; Manassas, USA) was grown in suspension cultures of HeLa cells (Ohio strain; Flow Laboratories; McLean, USA) for 40 h. HRV14 was prepared by polyethylene glycol precipitation and was re-suspended in PBS as described^40^.

### Culture of 16HBE14o-, a human bronchial epithelial cell line

The human bronchial epithelial cell line 16HBE14o- (Prof. D. C. Gruenert, University of California, San Francisco, USA) was used as a surrogate for the respiratory epithelium and compared with primary nasal epithelial cells. This cell line has previously been shown to retain the properties of differentiated airway epithelial cells. Cells grow in polarised monolayers, form tight junctions, apical microvilli and cilia and show regulated ion transport^14^. 16HBE14o- cells were grown as previously described. In short, cells were cultured in MEM containing 10% fetal bovine serum (FBS) (HyClone; GE Healthcare; Buckinghamshire, UK), 100 U/mL penicillin and 100 µg/mL streptomycin (Gibco; Thermo Fisher Scientific; Waltham, USA) at 37 °C in a humidified atmosphere containing 5% CO_2_. Cells were passaged at 70–90% confluence in collagen-fibronectin (BD Biosciences, San Jose, USA) coated tissue culture flasks.

### Tissue samples from patients and ethical considerations

Anonymised human nasal tissue samples from patients undergoing routine nasal surgery at the Department of Otorhinolaryngology (ORL) of the General Hospital of Vienna were used with the approval of the Ethics Committee of the Medical University of Vienna (EK Nr. 1476/2013). All methods were conducted in accordance with relevant guidelines and regulations of the Declaration of Helsinki. All patients gave written informed consent to donate their tissue before inclusion in the study. Male or female individuals aged between 18–65 years were included in the study (7 allergic, 13 non-allergic; average 38 years (21–55); 8 female, 12 male). We cultured cells of allergic and non-allergic individuals for the establishment of the cell culture models used in this work. Experiments performed to investigate the effect of various substances were done exclusively with cells from non-allergic individuals to exclude possible differences due to origin of cells from allergic and non-allergic individuals. Clinically relevant symptoms of allergy were recorded by ISAAC questionnaires and total IgE levels were determined by ImmunoCAP technology^41^. Additonally allergen-specific IgE levels were measured by allergen-chip microarrays (data not shown). Tissue samples of allergic patients were excluded from the experiments shown in this manuscript as this could have an influence on the results. We only used the cells of allergic and non-allergic individuals for the establishment of the cell culture models used in this work.

Patient samples were excluded if patients used nasal corticosteroids up to 2 months before undergoing surgery. Surgery was performed for anatomical reasons (septal deviation, turbinate hypertrophy) and apart from allergy none of the subjects had a history of a chronic or acute disease. The samples were stored in physiologic saline solution (0.9% NaCl) until surgery was completed, transferred to the laboratory on ice and processed immediately.

### Culture of primary human nasal epithelial cells

Tissue culture samples were washed with MEM medium including dithiothreitol (0.5 mg/mL DTT; Sigma-Aldrich, St. Louis, USA), desoxyribonuclease (10 µg/mL DNase; Sigma-Aldrich) and antibiotics [20 U/mL penicillin, 20 µg/mL streptomycin (Gibco; Thermo Fisher Scientific; Waltham, USA), 0.25 μg/mL amphotericin B (Sigma-Aldrich, St. Louis, USA), 50 μg/mL gentamicin (Gibco; Thermo Fisher Scientific)]. The nasal mucosa was then transferred to MEM medium containing antibiotics and protease (1% w/v; Sigma-Aldrich)/DNase (0.01% w/v; Sigma-Aldrich) mix. Cells were obtained by scraping the epithelial surface with a scalpel and were subsequently centrifuged. The cell pellet was re-suspended in MEM medium including a 0.25% (w/v) trypsin/EDTA mixture (Sigma-Aldrich) for 3 minutes. Cells were cultured in collagen/fibronectin coated flasks in bronchial epithelial growth medium (BEGM; BulletKit medium; Lonza Group LTD, Basel, Switzerland) at 37 °C and 5% CO_2_ until reaching confluence (7–10 days of culturing). Medium was changed every 2–3 days, as needed.

Cell types and morphological features of the cultured epithelium (21 days in ALI) were investigated by flow cytometry, immunohistological assessment and ciliary beat frequency measurements. Flow cytometry was performed using a FACSCanto II (BD Biosciences, San Jose, USA). Cells were stained for pan-cytokeratin (AE1/AE3, e488, eBioscience, San Diego, USA) to identify epithelial cells^42^ and were negative for CD45 (2D1, PE, eBioscience) to exclude other cells. Ciliary beat frequency measurement of the epithelial cell specimens was done by using a microscope (IX51, Olympus, Tokyo, Japan) equipped with a 100x oil immersion objective with a stage top incubator (HT200, Ibidi, Martinsried, Germany) as a heating system (37 °C) for life cell imaging. Images of ciliary activity were recorded at 300 frames per second with a high-speed coupled device camera (i-Speed 2, Olympus).

### Measurement of transepithelial resistance in the transwell system

Cells were transferred in collagen-fibronectin coated permeable 0.4 µm pore polyester transwell supports (Costar, Corning, New York, USA) at an ALI mimicking the physiological environment of the nasal epithelium. Cells were cultured in BEGM BulletKit medium (Lonza Group LTD). After reaching confluence, BEGM medium was removed from the upper well to establish ALI. The transwell system allowed transepithelial resistance (TER) measurement and penetration experiments. Resistance measurement using an Ohm-Volt meter (Merck Millipore; Millicell ERS-2, Darmstadt, Germany) was carried out at periodical intervals and epithelial barrier function was assessed. HDM, CSE or purified HRV14 were added to the upper chamber, while IFN-γ was added to the lower chamber. The concentration of the cytopathogenic agent HRV14 was determined by 50% tissue culture infective dose (TCID_50_) measurement. 150 and 1000 TCID_50_/cell concentrations of HRV14 were used to infect the confluent monolayers in MEM containing 1% FBS. In control wells mock infection was performed by adding PBS. TER was measured at indicated time points after treatment. In each case, at least 2 independent experiments were performed, each of them in duplicate or triplicate wells. Data are shown as mean values with standard errors of the mean (SEM) as error bars.

### Monitoring of barrier function with the xCELLigence real-time cell analysis system

Epithelial cells were sub-cultured from tissue culture flasks into collagen-fibronectin coated wells of E-plates 16 of the xCELLigence Real-Time Cell Analysis (RTCA) dual purpose (DP) system (ACEA Biosciences; San Diego, USA). Aliquots of 200 µL of cells were cultured in each well at a concentration of 1 × 10^5^ cells/mL. Real-time cell electronic sensing (RT-CES), a label-free technique for automatical and continuous electronic monitoring of adherent living cells was used^43,44^. The instrument allows non-invasive monitoring of impedance-based cell responses at physiological conditions which can be related to cell proliferation, morphology changes and cytotoxicity. Confluence of cell monolayers was ascertained by phase contrast microscopy (Olympus IX73) and corresponded to Cell Index values of 13–15 after approximately 24 hours of incubation, i.e. plateau phase. 16HBE14o- cells were consistently treated with the above-described substances (see: Measurement of TER) and agents after 24 hours of incubation and cell responses were monitored every 30 minutes. The last measuring point before adding the compounds was chosen as a normalisation time point. The Normalised Cell Index (NCI) amounts to 1 at this time point and changes of cell responses were shown from this value onwards. Cell Index is expressed as an arbitrary unit and is calculated from impedance measurements between cells and sensors of the xCELLigence system. NCI indicates relative values by comparison to the normalisation time point.

### Treatment of cells with Betamethasone or Fluticasone propionate

Betamethasone (3 µg/mL; Sigma-Aldrich) or Fluticasone propionate (2.5 µg/mL; Sigma-Aldrich) was added to cell monolayers 24 hours after seeding (i.e., when reaching the plateau phase). For control reasons, treatment of cells with the equal amount of PBS was performed. Three hours later the damaging substances were added to the wells and Cell Index values were measured throughout the experiments.

### Assessment of cytopathogenicity

Viability of cells was assessed by use of the crystal violet assay. 16HBE14o- cells were seeded in 96- well microtiter plates at a density of 1 × 10^5^ cells/mL and were incubated at 37 °C/5% CO_2_ until reaching confluence as previously described^12^. Cells were challenged with one of the following conditions: treatment with 2–32% CSE, infection with 150 or 1000 TCID_50_/cell HRV14, exposure to HDM extract (12.5, 150 or 200 µg/mL), treatment with IFN-γ (1, 5 or 20 ng/mL), treatment with Betamethasone (1.5, 3 or 6 µg/mL according to the protocols described above.

For the crystal violet assay cells were stained with 0.1% crystal violet in acetic acid/dH_2_O solution for 10 min at 21 °C after 48 h or 96 h of treatment with the respective substances (HDM, IFN-γ, CSE or HRV14). The crystal violet solution was removed and wells were gently washed with dH_2_O. Microtiter plates were visually analysed by phase contrast microscopy (Olympus IX73) and were photographed (iPhone, Apple Inc., USA).

Additionally a cell viability Kit based on a water soluble tetrazolium salt was used. Epithelial cells were seeded in a flat bottom 96-well plate. 24 hours later cells were treated in triplicates as described above with different concentrations of the respective substances HDM, IFN-γ, CSE, HRV14, Betamethasone or Fluticasone propionate. After 48 h or 96 h of treatment the TetraZ Cell Counting Kit (BioLegend, San Diego, California, USA) was applied. Absorbance was measured with Spark microplate reader at 450 nm (Tecan Group Ltd., Männedorf, Switzerland).

### Statistical analysis

For each time point separately, the mean value (i.e. mean TER or Cell Index) of each concentration was compared to the mean value of the untreated control by calculating two-sided Welch-tests. As the analyses are explorative, we did not adjust for multiple testing. Data are shown as mean values with SD or SEM as indicated.

Statistical analyses were conducted with R 3.3.2. The significance level has been set to alpha = 0.05.

## Electronic supplementary material

Supplementary Information

Supplementary Video
