# Supplementary material for: Betamethasone prevents human rhinovirus- and cigarette smoke- induced loss of respiratory epithelial barrier function
Source: Sci Rep. 2018 Jun 26;8:9688. doi: 10.1038/s41598-018-27022-y (PMC6018698; doi:10.1038/s41598-018-27022-y)
Supplement: Supplementary file 1 — Supplementary Information [file 41598_2018_27022_MOESM1_ESM.pdf]

Supplementary Information

Betamethasone prevents human rhinovirus- and cigarette smoke- induced loss of respiratory epithelial barrier function

Eva E. Waltl[1], Regina Selb[1], Julia Eckl-Dorna[1], Christian A. Mueller[1], Clarissa R. Cabauatan[2], Thomas Eiwegger[3,4,5], Yvonne Resch-Marat[2], Katarzyna Niespodziana[2], Susanne Vrtala[2], Rudolf Valenta[2], Verena Niederberger[1,]*

[1]Department of Otorhinolaryngology, Medical University of Vienna, Vienna, Austria
[2]Department of Pathophysiology and Allergy Research, Center for Pathophysiology, Infectiology and Immunology, Medical University of Vienna, Vienna, Austria
[3]Division of Immunology and Allergy, Food allergy and Anaphylaxis Program, The Department of Paediatrics, The Hospital for Sick Children, Toronto, Canada
[4]Research institute, The Hospital for Sick Children, Translational Medicine program, Toronto, Canada
[5]Department of Immunology, The University of Toronto, Toronto, Canada

*** Correspondence**
Verena Niederberger, MD, Assoc. Professor
Dept. of Otorhinolaryngology
Medical University of Vienna
AKH, 8.H1.12, Währinger Gürtel 18-20
A-1090 Vienna, Austria
E: verena.niederberger@meduniwien.ac.at
T: +43 1 40400 34380
F: +43 1 25 330338567

Supplementary Figures

Supplementary Figure S1. Morphological characterisation of cultured primary nasal epithelial cells. Giemsa staining of a cytospin preparation of a single cell from cultured primary nasal epithelial cells showing a columnar cell with apical cilia.

Supplementary Figure S2. Presence of allergens in house dust mite (*Dermatophagoides pteronyssinus*) extract. Allergens present in house dust mite (HDM) extract were detected with specific antisera directed against the HDM allergens Der p 1, Der p 2, Der p 5, Der p 7, Der p 10, Der p 21 and Der p 23. Normal rabbit serum (P) and buffer control (BC) served as negative controls. The molecular weight marker is shown in kilo Daltons (kDa).

Supplementary Figure S3. Cytopathogenicity of factors tested for effects on barrier function. 16HBE14o- cells cultured in 96-well-plates were treated with different concentrations of house dust mite extract **(A)**, interferon-γ **(B)**, cigarette smoke extract **(C)**, human rhinovirus 14 **(D)** or betamethasone **(E)** compared to medium control **(A-E)**. After 48 **(C, D)** or 96 hours **(A, B, E)** cells were stained with crystal violet solution. Viable cells are stained blue while decolouration of wells indicates cytotoxicity.

Supplementary Figure S4. Fluticasone propionate does not prevent cigarette smoke- and rhinovirus-induced damage of respiratory epithelial cells. 16HBE14o- cells were incubated at time point 0 with fluticasone propionate (2.5 µg/mL) or medium. Three hours thereafter, either 4 % cigarette smoke extract **(A)** was added to

the cells or cells were infected with 150 $TCID_{50}$/cell of rhinovirus 14 **(B)**. Data were normalised at time point 0 and impedance values (y-axis: normalised Cell Index) were measured every 30 minutes for 48 hours. Results from three independent experiments performed in duplicates are shown. Statistical significance analyses were performed with the two-sided Welch-tests. Standard errors of the mean values are visualised as error bars.

Supplementary Figure S5. Assessment of cell viability after addition of different factors. 16HBE14o- cells were treated with different concentrations of Fluticasone propionate, Betamethasone, interferon-γ, house dust mite extract, cigarette smoke extract or human rhinovirus 14 compared to medium alone (untreated, negative control) or ethanol (positive control). Mean values with SD of triplicates are shown. The box indicates the SD of the untreated cells.

Supplementary Tables I-V. For each Figure (column 1) and time point (column 2) the mean value (mean TER or Cell Index) of the first parameter was compared to the mean value of the second parameter (column 3) by calculating two-sided Welch-tests. Statistical analyses were conducted with R 3.3.2. The significance level has been set to alpha = 0.05 (highlighted in yellow). Time = hours (see Figures); Comparison = Comparison of first parameter with (vs) second parameter (column 3); Estimate = mean group differences (mean value of tested concentrations – mean value of untreated control) and 95% confidence interval for mean group difference (column 4); p = unadjusted p-value (Welch-test) (column 5). $P<0.05$ highlighted in yellow.

Supplementary Movie. Microscopic video analysis of a representative sample of cultured primary nasal epithelial cells showing the exhibition of normal healthy ciliary beat activity and frequency.

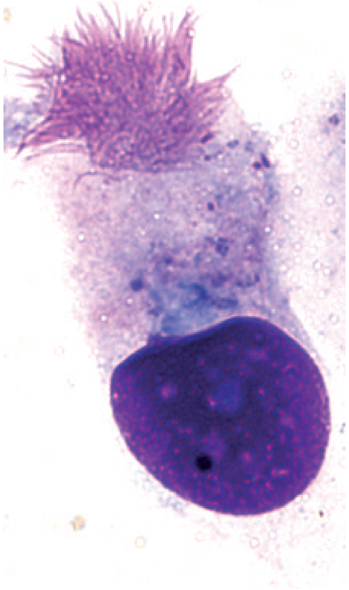

Supplementary Figure S1

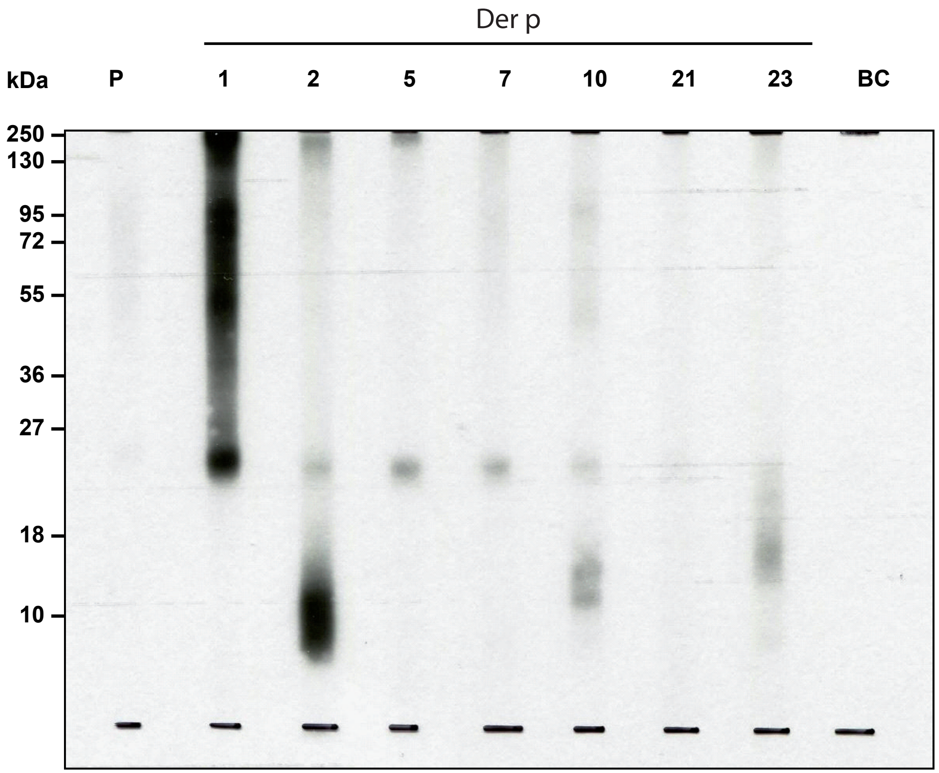

Der p	Biochemical function	MW (kDa) SDS-PAGE
1	Cysteine protease	25
2	ML domain lipid binding protein	15
5	Unknown	15
7	Lipid-binding protein	26, 29, 31
10	Tropomyosin	37
21	Unknown	15
23	Chitin-binding protein	14

Supplementary Figure S2

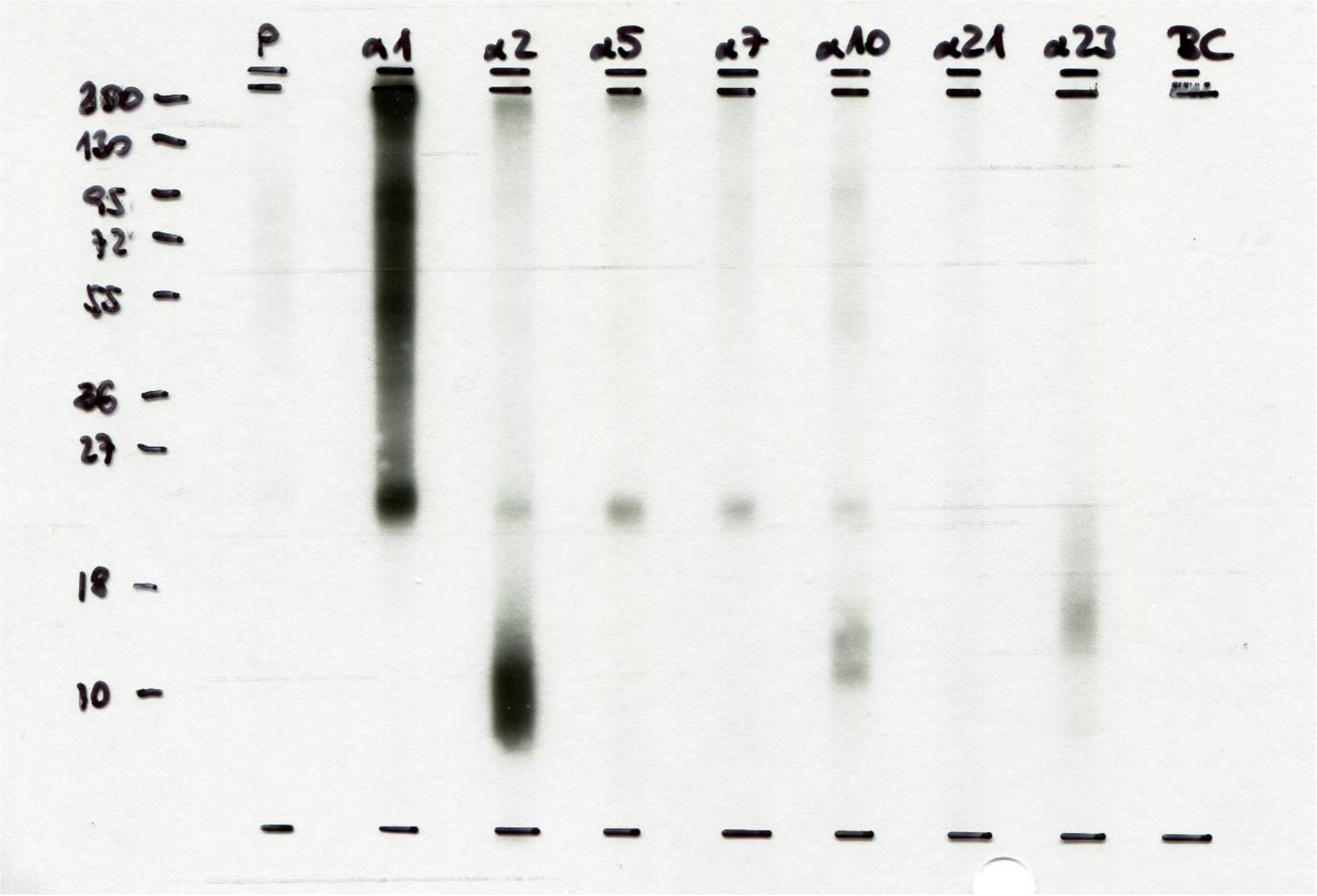

Supplementary Figure S2; Full-length blot

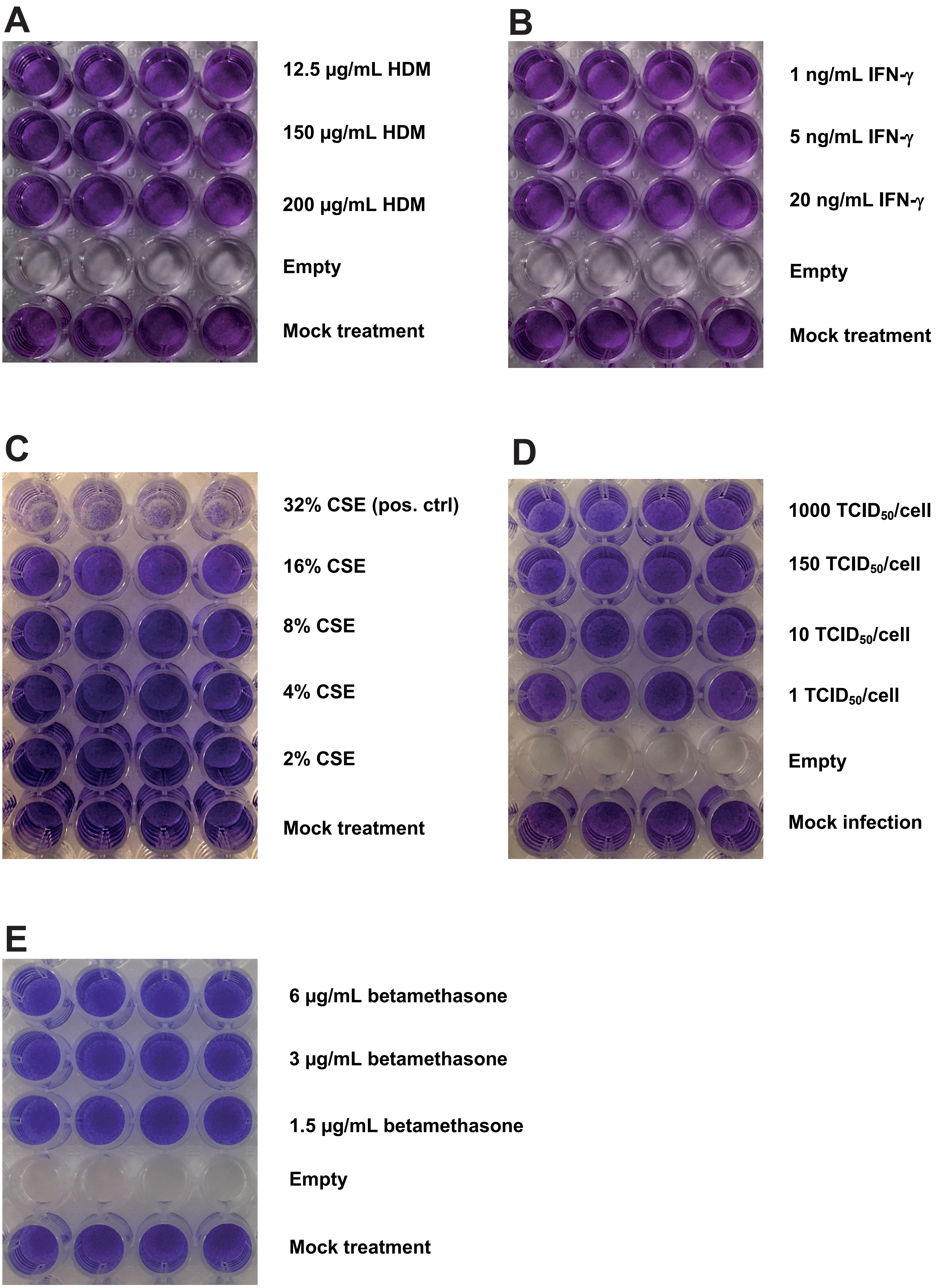

Supplementary Figure S3

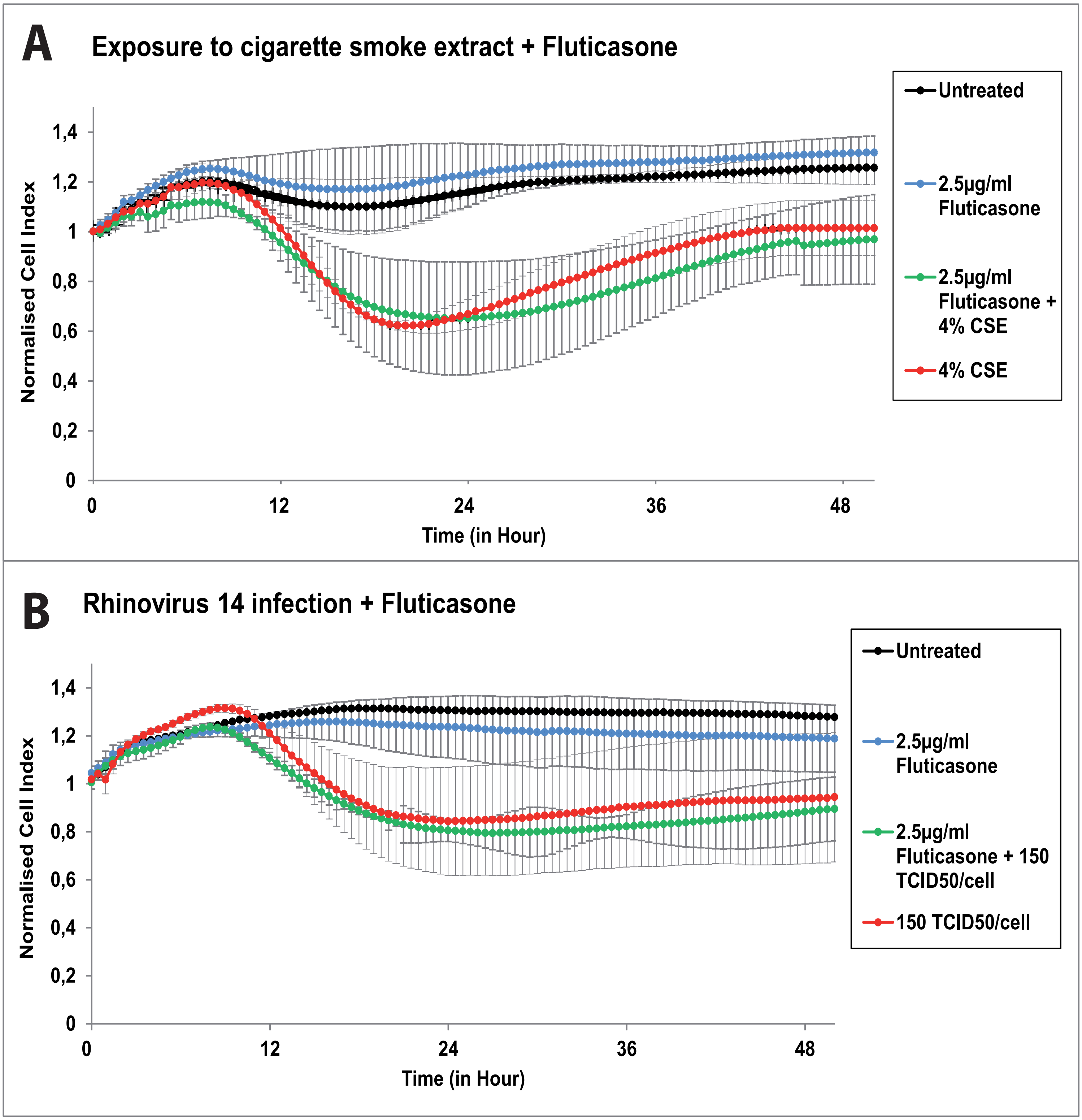

A
Exposure to cigarette smoke extract + Fluticasone
Untreated
2.5μg/ml Fluticasone
2.5μg/ml Fluticasone + 4% CSE
4% CSE
Normalised Cell Index
1,4
1,2
1
0,8
0,6
0,4
0,2
0
0
12
24
36
48
Time (in Hour)
B
Rhinovirus 14 infection + Fluticasone
Untreated
2.5μg/ml Fluticasone
2.5μg/ml Fluticasone + 150 TCID50/cell
150 TCID50/cell
Normalised Cell Index
1,4
1,2
1
0,8
0,6
0,4
0,2
0
0
12
24
36
48
Time (in Hour)

Figure S4

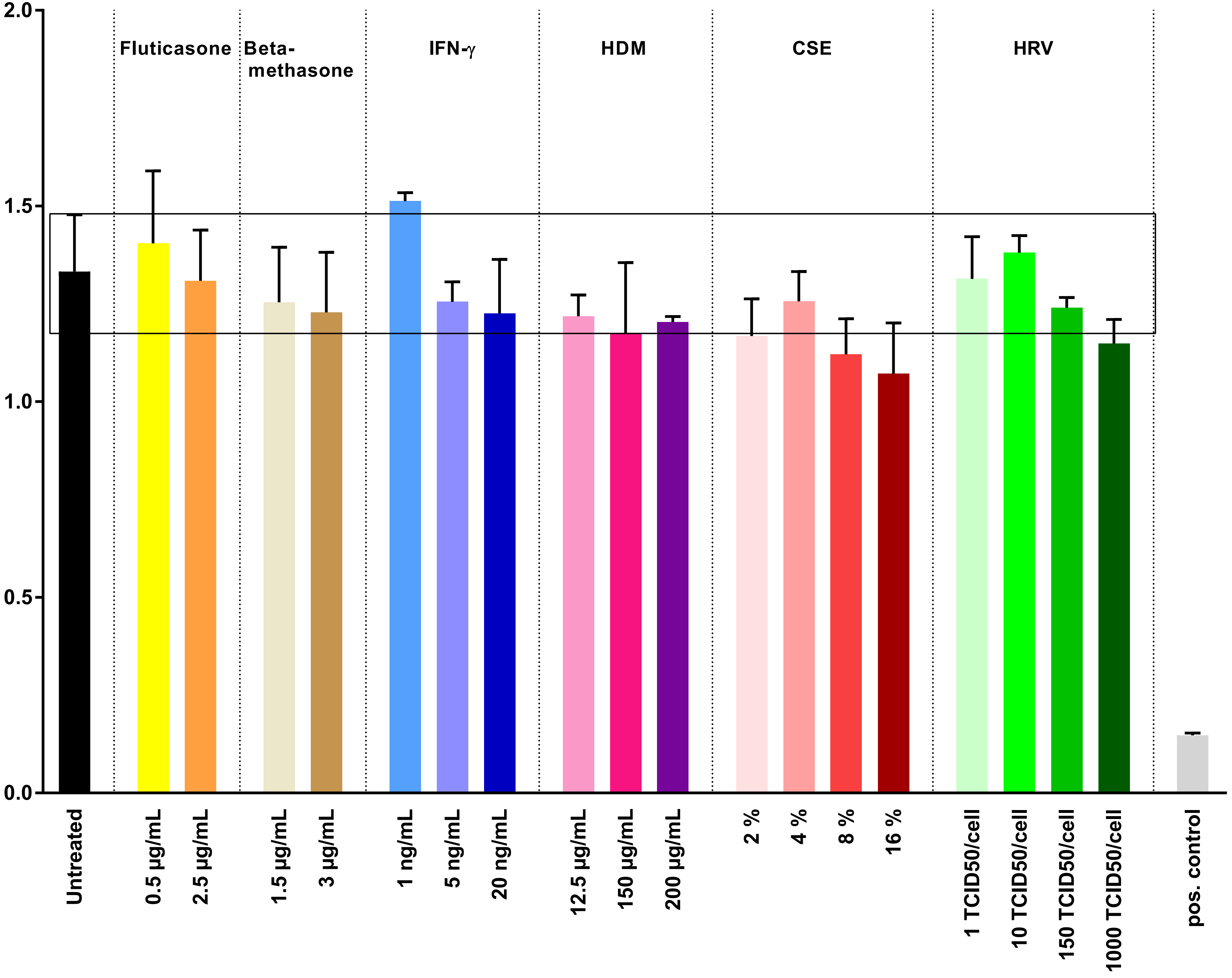
2.0
1.5
1.0
0.5
0.0
Fluticasone
Beta-
methasone
IFN-γ
HDM
CSE
HRV
Untreated
0.5 µg/mL
2.5 µg/mL
1.5 µg/mL
3 µg/mL
1 ng/mL
5 ng/mL
20 ng/mL
12.5 µg/mL
150 µg/mL
200 µg/mL
2 %
4 %
8 %
16 %
1 TCID50/cell
10 TCID50/cell
150 TCID50/cell
1000 TCID50/cell
pos. control

Figure S5

Supplementary TABLE I. Statistical analyses of Figure 1

Figure 1A upper	Time	Comparison	Estimate	p
	0	12.5 vs 0	-67[-1422,1288]	0,9
	0	150 vs 0	200[-1295,1695]	0,66
	0	200 vs 0	183[-1393,1759]	0,68
	24	12.5 vs 0	0[-1392,1392]	1
	24	150 vs 0	400[-1520,2320]	0,47
	24	200 vs 0	1850[1105,2595]	0,0064
	48	12.5 vs 0	-83[-1535,1368]	0,86
	48	150 vs 0	-33[-796,729]	0,88
	48	200 vs 0	50[-1506,1606]	0,92
	72	12.5 vs 0	0[-1144,1144]	1
	72	150 vs 0	-733[-2061,595]	0,14
	72	200 vs 0	-642[-1780,496]	0,16
	96	12.5 vs 0	-200[-1037,637]	0,54
	96	150 vs 0	-2042[-2854,-1229]	0,0084
	96	200 vs 0	-2772[-3527,-2016]	0,0005
**Figure 1A lower**	24	12.5 vs 0	-0.131[-0.412,0.15]	0,187
	24	150 vs 0	0.058[-0.852,0.968]	0,811
	24	200 vs 0	-0.468[-1.333,0.396]	0,145
	48	12.5 vs 0	-0.147[-0.319,0.025]	0,076
	48	150 vs 0	-0.103[-0.334,0.128]	0,276
	48	200 vs 0	-0.64[-0.817,-0.462]	0,0006
	72	12.5 vs 0	0.084[-0.073,0.241]	0,177
	72	150 vs 0	-0.334[-0.487,-0.181]	0,0042
	72	200 vs 0	-0.619[-0.803,-0.436]	0,0047
	96	12.5 vs 0	0.086[-0.152,0.323]	0,305
	96	150 vs 0	-0.611[-0.882,-0.341]	0,01
	96	200 vs 0	-0.611[-0.882,-0.341]	0,01
**Figure 1B upper**	0	1 vs 0	-333[-1281,614]	0,361
	0	5 vs 0	-300[-1152,552]	0,369
	0	20 vs 0	-433[-1570,703]	0,313
	24	1 vs 0	867[-444,2177]	0,138
	24	5 vs 0	133[-1021,1288]	0,694
	24	20 vs 0	508[-1595,2612]	0,493
	48	1 vs 0	-1058[-2458,341]	0,089
	48	5 vs 0	-1125[-2414,164]	0,07
	48	20 vs 0	-667[-2322,988]	0,321
	72	1 vs 0	900[-559,2359]	0,156
	72	5 vs 0	-633[-4234,2967]	0,573
	72	20 vs 0	-750[-1954,454]	0,121
	96	1 vs 0	-258[-1712,1195]	0,603
	96	5 vs 0	-475[-2856,1906]	0,584
	96	20 vs 0	-2167[-3609,-725]	0,015
**Figure 1B lower**	24	1 vs 0	-0.024[-0.199,0.152]	0,705

	24	5 vs 0	0.103[-0.342,0.548]	0,456
	24	20 vs 0	0.267[-0.388,0.923]	0,232
	48	1 vs 0	0.024[-0.16,0.207]	0,736
	48	5 vs 0	0.093[-0.413,0.599]	0,563
	48	20 vs 0	0.074[-0.107,0.254]	0,321
	72	1 vs 0	-0.395[-0.867,0.076]	0,076
	72	5 vs 0	-0.362[-0.634,-0.09]	0,027
	72	20 vs 0	-0.52[-0.794,-0.247]	0,012
	96	1 vs 0	-0.102[-0.52,0.316]	0,534
	96	5 vs 0	-0.193[-0.611,0.224]	0,267
	96	20 vs 0	-0.817[-1.391,-0.243]	0,019
**Figure 1C upper**	0	2 vs 0	150[-937,1237]	0,72
	0	8 vs 0	633[-950,2217]	0,31
	0	16 vs 0	117[-1080,1313]	0,727
	12	2 vs 0	467[-1121,2054]	0,417
	12	8 vs 0	833[-3084,4751]	0,484
	12	16 vs 0	1483[-2678,5645]	0,283
	24	2 vs 0	217[-4768,5202]	0,886
	24	8 vs 0	2133[390,3877]	0,027
	24	16 vs 0	1867[-2090,5824]	0,214
	36	2 vs 0	467[-1774,2708]	0,488
	36	8 vs 0	1233[-2706,5173]	0,317
	36	16 vs 0	-1317[-3153,520]	0,096
	48	2 vs 0	525[-1160,2210]	0,405
	48	8 vs 0	1425[-501,3351]	0,089
	48	16 vs 0	-2058[-3746,-370]	0,03
**Figure 1C lower**	12	2 vs 0	-0.118[-0.44,0.204]	0,335
	12	8 vs 0	-0.286[-0.627,0.055]	0,073
	12	16 vs 0	-0.981[-1.341,-0.621]	0,0057
	24	2 vs 0	-0.033[-0.522,0.456]	0,859
	24	8 vs 0	-0.475[-0.977,0.028]	0,059
	24	16 vs 0	-0.994[-1.56,-0.428]	0,017
	36	2 vs 0	0.054[-0.298,0.406]	0,638
	36	8 vs 0	-0.485[-1.054,0.083]	0,069
	36	16 vs 0	-0.825[-0.984,-0.667]	0,0016
	48	2 vs 0	0.053[-0.243,0.349]	0,567
	48	8 vs 0	-0.463[-1.028,0.103]	0,073
	48	16 vs 0	-0.753[-0.85,-0.655]	0,00049
**Figure 1D upper**	0	150 vs 0	-58[-147,30]	0,135
	0	1000 vs 0	-33[-105,38]	0,184
	12	150 vs 0	-1867[-5194,1460]	0,181
	12	1000 vs 0	-3117[-6770,537]	0,069
	24	150 vs 0	-3333[-4418,-2248]	0,0011
	24	1000 vs 0	-6370[-7888,-4852]	0,0005
	36	150 vs 0	-2533[-4062,-1005]	0,011
	36	1000 vs 0	-5248[-7529,-2968]	0,0058

	48	150 vs 0	-1867[-4731,998]	0,109
	48	1000 vs 0	-4749[-7278,-2220]	0,0066
**Figure 1D lower**	12	150 vs 0	-0.118[-0.279,0.043]	0,111
	12	1000 vs 0	-0.17[-0.327,-0.014]	0,040
	24	150 vs 0	-0.071[-0.305,0.162]	0,356
	24	1000 vs 0	-0.164[-0.379,0.05]	0,093
	36	150 vs 0	-0.034[-0.272,0.205]	0,702
	36	1000 vs 0	-0.181[-0.46,0.099]	0,112
	48	150 vs 0	-0.053[-0.33,0.223]	0,596
	48	1000 vs 0	-0.204[-0.534,0.126]	0,119

Supplementary TABLE II. Statistical analyses of Figure 2

Figure 2A upper	Time	Comparison	Estimate	p
	12	12.5 vs 0	-0.081[-0.33,0.169]	0,386
	12	150 vs 0	-0.095[-0.427,0.238]	0,414
	12	200 vs 0	-0.244[-0.68,0.191]	0,155
	24	12.5 vs 0	-0.131[-0.412,0.15]	0,187
	24	150 vs 0	0.058[-0.852,0.968]	0,811
	24	200 vs 0	-0.468[-1.333,0.396]	0,145
	36	12.5 vs 0	-0.127[-0.272,0.017]	0,071
	36	150 vs 0	0.032[-0.669,0.734]	0,870
	36	200 vs 0	-0.587[-0.999,-0.176]	0,023
	48	12.5 vs 0	-0.147[-0.319,0.025]	0,076
	48	150 vs 0	-0.103[-0.334,0.128]	0,276
	48	200 vs 0	-0.64[-0.817,-0.462]	0,00060
**Figure 2A lower**	12	12.5 vs 0	-0.092[-0.285,0.101]	0,232
	12	150 vs 0	-0.151[-0.607,0.305]	0,354
	12	200 vs 0	-0.348[-0.868,0.172]	0,116
	24	12.5 vs 0	-0.132[-0.414,0.149]	0,215
	24	150 vs 0	-0.201[-0.554,0.152]	0,184
	24	200 vs 0	-0.616[-1.905,0.673]	0,189
	36	12.5 vs 0	-0.161[-0.412,0.089]	0,142
	36	150 vs 0	-0.209[-0.631,0.213]	0,221
	36	200 vs 0	-0.778[-1.967,0.412]	0,112
	48	12.5 vs 0	-0.154[-0.471,0.163]	0,229
	48	150 vs 0	-0.207[-0.544,0.13]	0,135
	48	200 vs 0	-0.958[-1.636,-0.28]	0,021
**Figure 2B upper**	48	1 vs 0	0.024[-0.16,0.207]	0,736
	48	5 vs 0	0.093[-0.413,0.599]	0,563
	96	1 vs 0	-0.102[-0.52,0.316]	0,534
	96	5 vs 0	-0.193[-0.611,0.224]	0,267
	144	1 vs 0	-0.082[-0.373,0.209]	0,472

	144	5 vs 0	-0.369[-0.783,0.045]	0,066
	192	1 vs 0	-0.209[-0.998,0.581]	0,497
	192	5 vs 0	-0.566[-1.228,0.096]	0,069
	240	1 vs 0	-0.177[-1.067,0.712]	0,606
	240	5 vs 0	-0.464[-1.305,0.378]	0,146
**Figure 2B lower**	48	1 vs 0	-0.074[-0.519,0.37]	0,655
	48	5 vs 0	-0.083[-0.585,0.418]	0,668
	96	1 vs 0	-0.064[-0.477,0.349]	0,679
	96	5 vs 0	-0.049[-0.761,0.664]	0,847
	144	1 vs 0	-0.499[-1.64,0.641]	0,240
	144	5 vs 0	-1.118[-1.627,-0.609]	0,010
	192	1 vs 0	-1.033[-1.465,-0.6]	0,0073
	192	5 vs 0	-1.093[-1.568,-0.618]	0,010
	240	1 vs 0	-0.582[-1.857,0.693]	0,189
	240	5 vs 0	-0.588[-1.864,0.687]	0,185
**Figure 2C upper**	12	2 vs 0	-0.118[-0.44,0.204]	0,335
	12	8 vs 0	-0.286[-0.627,0.055]	0,073
	24	2 vs 0	-0.033[-0.522,0.456]	0,859
	24	8 vs 0	-0.475[-0.977,0.028]	0,059
	36	2 vs 0	0.054[-0.298,0.406]	0,638
	36	8 vs 0	-0.485[-1.054,0.083]	0,069
	48	2 vs 0	0.053[-0.243,0.349]	0,567
	48	8 vs 0	-0.463[-1.028,0.103]	0,073
**Figure 2C lower**	12	2 vs 0	-0.434[-0.96,0.092]	0,076
	12	8 vs 0	-0.88[-1.124,-0.636]	0,00057
	24	2 vs 0	-0.256[-0.586,0.073]	0,086
	24	8 vs 0	-0.759[-1.191,-0.328]	0,014
	36	2 vs 0	-0.143[-0.384,0.098]	0,153
	36	8 vs 0	-0.451[-1.072,0.17]	0,092
	48	2 vs 0	-0.088[-0.402,0.225]	0,397
	48	8 vs 0	-0.226[-0.801,0.349]	0,246
**Figure 2D upper**	12	150 vs 0	-0.118[-0.279,0.043]	0,111
	12	1000 vs 0	-0.17[-0.327,-0.014]	0,040
	24	150 vs 0	-0.071[-0.305,0.162]	0,356
	24	1000 vs 0	-0.164[-0.379,0.05]	0,093
	36	150 vs 0	-0.034[-0.272,0.205]	0,702
	36	1000 vs 0	-0.181[-0.46,0.099]	0,112
	48	150 vs 0	-0.053[-0.33,0.223]	0,596
	48	1000 vs 0	-0.204[-0.534,0.126]	0,119
**Figure 2D lower**	12	150 vs 0	-0.26[-0.542,0.021]	0,062
	12	1000 vs 0	-0.512[-1.198,0.175]	0,093
	24	150 vs 0	-0.571[-1.011,-0.131]	0,030
	24	1000 vs 0	-0.993[-1.227,-0.759]	0,0016
	36	150 vs 0	-0.488[-1.265,0.289]	0,117
	36	1000 vs 0	-0.984[-1.209,-0.76]	0,0011
	48	150 vs 0	-0.462[-1.308,0.384]	0,144

	48	1000 vs 0	-0.97[-1.196,-0.744]	0,0023

Supplementary TABLE III. Statistical analyses of Figure 3

Figure 3	Time	Comparison	Estimate	p
	14	CSE vs 0	-0.106[-0.286,0.075]	0,174
	14	HDM vs 0	0.092[-0.041,0.224]	0,114
	14	combination vs 0	-0.217[-0.378,-0.056]	0,020
	26	CSE vs 0	-0.07[-0.808,0.668]	0,775
	26	HDM vs 0	-0.006[-0.343,0.331]	0,957
	26	combination vs 0	-0.364[-0.861,0.133]	0,107
	38	CSE vs 0	0.105[-0.112,0.321]	0,244
	38	HDM vs 0	-0.062[-0.354,0.23]	0,553
	38	combination vs 0	-0.275[-1.117,0.568]	0,316
	50	CSE vs 0	0.198[-0.085,0.482]	0,099
	50	HDM vs 0	-0.057[-0.207,0.094]	0,294
	50	combination vs 0	-0.153[-0.595,0.289]	0,284

Supplementary TABLE IV. Statistical analyses of Figure 4

Figure 4A	Time	Comparison	Estimate	p
	14	0 vs CSE	0.25[-0.09,0.591]	0.11
	14	Betamethasone vs CSE	0.31[-0.134,0.755]	0.117
	14	combination vs CSE	0.16[-0.107,0.428]	0.161
	26	0 vs. CSE	0.514[0.31,0.717]	0.0042
	26	Betamethasone vs CSE	0.555[0.353,0.757]	0.00199
	26	combination vs CSE	0.252[-0.136,0.639]	0.131
	38	0 vs CSE	0.271[-0.168,0.71]	0.121
	38	Betamethasone vs CSE	0.343[-0.108,0.795]	0.082
	38	combination vs CSE	0.238[-0.155,0.63]	0.167
	50	0 vs CSE	0.243[-0.17,0.657]	0.142
	50	Betamethasone vs CSE	0.295[-0.085,0.676]	0.092
	50	combination vs CSE	0.275[-0.264,0.814]	0.221
**Figure 4B**	14	0 vs HRV	0.08[-0.494,0.654]	0.658
	14	Betamethasone vs HRV	0.145[-0.502,0.793]	0.437
	14	combination vs HRV	0.239[-0.313,0.792]	0.251
	26	0 vs HRV	0.375[-0.326,1.076]	0.151
	26	Betamethasone vs HRV	0.342[-0.333,1.016]	0.171
	26	combination vs HRV	0.213[-0.584,1.009]	0.490
	38	0 vs HRV	0.398[-0.098,0.895]	0.075

	38	Betamethasone vs HRV	0.303[-0.141,0.747]	0.109
	38	combination vs HRV	0.249[-0.355,0.854]	0.302
	50	0 vs HRV	0.392[0.098,0.686]	0.022
	50	Betamethasone vs HRV	0.27[-0.039,0.58]	0.072
	50	combination vs HRV	0.352[0.063,0.642]	0.029

Supplementary TABLE V. Statistical analyses of Figure S4

Figure S4A	Time	Comparison	Estimate	p
	14	0 vs CSE	0.249[-0.219,0.717]	0.203
	14	Fluticasone vs CSE	0.356[-0.072,0.784]	0.081
	14	combination vs CSE	0.002[-0.513,0.516]	0.993
	26	0 vs CSE	0.447[0.161,0.734]	0.018
	26	Fluticasone vs CSE	0.505[0.17,0.839]	0.014
	26	combination vs CSE	-0.075[-0.375,0.226]	0.529
	38	0 vs CSE	0.271[-0.013,0.555]	0.055
	38	Fluticasone vs CSE	0.335[0.066,0.604]	0.029
	38	combination vs CSE	-0.076[-0.86,0.709]	0.757
	50	0 vs CSE	0.227[-0.08,0.533]	0.109
	50	Fluticasone vs CSE	0.27[0.003,0.538]	0.049
	50	combination vs CSE	-0.06[-0.755,0.635]	0.786
**Figure S4B**	14	0 vs HRV	0.087[-0.776,0.95]	0.769
	14	Fluticasone vs HRV	0.209[-0.233,0.651]	0.235
	14	combination vs HRV	0.009[-0.455,0.474]	0.949
	26	0 vs HRV	0.457[-0.396,1.309]	0.174
	26	Fluticasone vs HRV	0.386[-0.445,1.216]	0.229
	26	combination vs HRV	-0.053[-0.861,0.756]	0.856
	38	0 vs HRV	0.388[-0.557,1.334]	0.279
	38	Fluticasone vs HRV	0.295[-0.719,1.31]	0.380
	38	combination vs HRV	-0.082[-1.045,0.881]	0.795
	50	0 vs HRV	0.339[-0.636,1.315]	0.344
	50	Fluticasone vs HRV	0.253[-0.713,1.218]	0.468
	50	combination vs HRV	-0.049[-1.137,1.039]	0.875
